# Variants and haplotypes in Flap endonuclease 1 and risk of gallbladder cancer and gallstones: a population-based study in China

**DOI:** 10.1038/srep18160

**Published:** 2015-12-15

**Authors:** Xingyuan Jiao, Ying Wu, Liansuo Zhou, Jinyun He, Chonghua Yang, Peng Zhang, Ronglin Hu, Canqiao Luo, Jun Du, Jian Fu, Jinsen Shi, Rui He, Dongming Li, Wang Jun

**Affiliations:** 1 Department of General Surgery, The First Affiliated Hospital, Sun Yat-Sen University, Guangzhou 510080, China; 2 Department of General Surgery and Transplantation Surgery, University Hospital Duisburg-Essen, D-45122, Germany; 3 Department of Biostatistics, The First Affiliated Hospital, Sun Yat-Sen University, Guangzhou 510080, China; 4 Department of General Surgery, The First Affiliated Hospital, Xian Medical College, Xian 710061, China; 5 Deparment of Pathology, Sun Yat-Sen University School of Medicine, Guangzhou 510080, China; 6 Department of Molecular Biology, Sun Yat-Sen University School of Pharmacy, Guangzhou 510080, China; 7 Department of Hepatobiliary Surgery, The First Affiliated Hospital, Xian Jiaotong University, Xian 710061, China; 8 Department of Anatomy, Shenzhen University School of Medicine, Shenzhen 518060, China

## Abstract

The role of FEN1 genetic variants on gallstone and gallbladder cancer susceptibility is unknown. FEN1 SNPs were genotyped using the polymerase chain reaction-restriction fragment length polymorphism method in blood samples from 341 gallbladder cancer patients and 339 healthy controls. The distribution of FEN1-69G > A genotypes among controls (AA, 20.6%; GA, 47.2% and GG 32.2%) was significantly different from that among gallbladder cancer cases (AA, 11.1%; GA, 48.1% and GG, 40.8%), significantly increased association with gallbladder cancer was observed for subjects with both the FEN1-69G > A GA (OR = 1.73, 95% CI = 1.01–2.63) and the FEN1-69G > A GG (OR = 2.29, 95% CI = 1.31–3.9). The distribution of FEN1 -4150T genotypes among controls (TT, 21.8%;GT, 49.3% and GG 28.9%) was significantly different from that among gallbladder cancer cases (TT, 12.9%; GT, 48.4% and GG 38.7%), significantly increased association with gallbladder cancer was observed for subjects with both the FEN1-4150T GT(OR = 1.93, 95% CI = 1.04–2.91) and the FEN1-4150T GG(OR = 2.56, 95% CI = 1.37–5.39). A significant trend towards increased association with gallbladder cancer was observed with potentially higher-risk FEN1-69G > A genotypes (P < 0.001, χ2 trend test) and FEN14150G > T (P < 0.001, χ2 trend test) in gallstone presence but not in gallstone absence (P = 0.81, P = 0.89, respectively). In conclusion, this study revealed firstly that *FEN1* polymorphisms and haplotypes are associated with gallbladder cancer risk.

Gallbladder cancer (GBC) is a relatively uncommon malignancy, but it has an unique geographical distribution in the world, with the highest incidence rate occurring in Chile, Poland, India, Japan, and Israel, and gallbladder cancer also occurs more common in certain ethnic population, such as Native American Indians and Hispanics^1^,^2^,^3^,^4^. In China, gallbladder cancer is relatively rare; however, its incidence has increased in the past several decades^5^,^6^. The pathogenesis of gallbladder cancer is not completely understood but a multifactorial etiology may be responsible for its development^7^,^8^. The risk factors for gallbladder cancer include female gender, obesity, chronic cholecystitis, cholelithiasis (gallstones) exposure to specific environmental chemicals (often occupational) and chronic bacterial infections of the gallbladder^9^,^10^. A recent study showed that single nucleotide polymorphisms (SNPs) of various genes are associated with gallbladder cancer risk^11^. As in other tissues, these risk factors contribute to the development of gallbladder cancer through multiple genetic alterations that activate oncogenes and silence tumor suppressor genes^7^. When carcinogens or other environmental factors alter DNA structure or modify DNA bases, DNA repair proteins repair the damage and maintain DNA integrity; however, alterations in the cell’s DNA repair proteins may reduce the cell’s ability to repair damaged DNA. The reduction or loss off a cell’s DNA damage repair capacity will lead to genetic alterations in that cell^11^. Four major DNA repair pathways have been identified to be responsible for repairing damaged DNA in cells. These pathways are the base excision, nucleotide excision (NER), double-strand break, and mismatch repair pathways^12^. Research on DNA repair and the degree to which SNPs effect the function of important DNA repair genes could help identify genetic risk factors of gallbladder cancer development and help develop novel strategies for gallbladder cancer treatment.

To this end, we have studied the association of flap endonuclease 1 (FEN1; MIM 600393) SNPs with gallbladder cancer. FEN1 is multi-functional nuclease, and is involved in DNA base-excision repair (BER) and DNA replication. FEN1 efficiently removes the 5′-flap during long-patch base-excision repair and processes Okazaki fragments during DNA replication^13^,^14^. Additionally, FEN1 promotes DNA fragmentation in apoptotic cells by acting as a 5′ exonuclease and a gap-dependent endonuclease^15^,^16^. Through these processes, FEN1 plays an essential role in the maintenance of genomic stability and protects against malignant transformation^17^. A previous study showed that yeast with functionally impaired FEN1 (RAD27 in yeast) had a remarkably increased rate of spontaneous mutation of genomic DNA^18^. In mice, haplo-insufficient FEN1 led to increased genome instability and carcinogenesis^19^, and *FEN1* mutations in transgenic mice reduced nuclease activity and promoted cancer development in multiple organs^20^. Naturally occurring genetic variations in FEN1 expression or function may also contribute to cancer susceptibility^21^. *FEN1* -69G > A (rs174538, in the *FEN1* promoter region) and 4150G > T (rs4246215, in the *FEN1* 3′-untranslated region) SNPs are associated with elevated risks of breast cancer^22^, lung cancer^23^, hepatocellular carcinoma^24^, esophageal cancer^25^, gastric cancer^26^ and glioma^27^. Thus, we hypothesized that gallbladder cancer risk would be synergistically increased for the interaction of genetic variants with environmental factors (e.g. gallstones). We assessed the association of two functional *FEN1* SNPs and their haplotypes with gallstone and gallbladder cancer risk in a Chinese population.

## Material and Methods

### Study subjects

We used blood samples from 341 gallbladder cancer patients and 339 healthy controls. This cohort was previously used to assess the association of xeroderma pigmentosum, complementation group C (XPC) Ala499Val (C > T) and Lys939Gln (A > C) polymorphisms with gallbladder cancer risk^28^. The cohort was composed of subjects who were residents of different geographic regions in China. Between January 2006 and December 2013, gallbladder cancer patients were recruited from four different hospitals (The First Affiliated Hospital of Sun Yat-Sen University, The First Affiliated Hospital of Xian Jiaotong University, The Second Affiliated Hospital of Guangzhou Medical University and the First Affiliated Hospital of Xian Medical College). Pathologists from the Pathology Department at the Sun Yat-Sen University School of Medicine histologically confirmed that all the cases in the cohort were gallbladder adenocarcinomas. We excluded patients with the following risk factors from this study: occupational exposure to ultraviolet radiation, occupational exposure to chemical carcinogens, chronic bacterial infections, carrier-state typhoid fever or ulcerative colitis. The control patients were recruited from the same four hospitals and were age and gender matched to the gallbladder cancer patients. Prior to inclusion in the study, the control patients were subjected to a routine health examination and were found to be free of cancer. Before participating our study, all subjects filled out a detailed questionnaire that included questions concerning patient demographics. Clinicopathological data from gallbladder cancer patients and healthy controls were also obtained. Since gallstones are an established gallbladder cancer risk factor (a risk that increases with increasing gallstone size)^29^,^30^, we recruited a panel of specialists to record the presence and size of gallstones using real-time ultrasound examination of all patients recruited for this study. Gallstones were identified based on the presence of movable hyperechoic foci casting acoustic shadows and measured using a PAV Electronic Sliding Caliper, Type Classic 6511. A pilot study was performed on 100 randomly selected healthy subjects who were not included in this study cohort. To assess inter-observer reliability, the Kappa value for gallstone diagnosis between specialists was 0.85 [95% confidence interval (CI): 0.74–0.95]. This study was approved by the Institution Review Board of the Ethics Committees of all participating institutes (The First Affiliated Hospital of Sun Yat-Sen University, Sun Yat-Sen University School of Medicine, Sun Yat-Sen University School of Pharmacy, The First Affiliated Hospital of Xian Jiaotong University, The Second Affiliated Hospital of Guangzhou Medical University, The First Affiliated Hospital of Xian Medical College, Shenzhen University School of Medicine, China. University Hospital Duisburg-Essen, Germany) and an informed consent form was obtained from each participant before the collection of blood samples and clinical evaluations. The methods were carried out in accordance with the approved guidelines.

### Genotyping of FEN1 polymorphism

Genomic DNA was extracted from the peripheral blood of each participant. In brief, the buffy coat fraction from 4.9 ml venous blood in ethylenediaminetetraacetic acid was isolated and genomic DNA was extracted using a standard phenol-chloroform procedure^31^. FEN1 -69G/A (rs174538: G > A; NM_004111.4) and 4150G/T (rs4246215: G > T; NM_004111.4) SNPs were genotyped using a previously described polymerase chain reaction (PCR) restriction fragment length polymorphism assay^23^,^31^. PCR primers were designed based on the Genbank reference sequence: 5′-ggaggttccaggagcgtcta-3′ and 5′-ttctccaccgcttgtccc-3′ for FEN1-69G > A; 5′-tatgtcaggctcaaaccac-3′ and 5′-cagccagtaatcagtcacaa-3′ for FEN1 50G > T. PCR amplification was performed using a 25 μL reaction mixture containing 100 ng DNA, 0.1 mmol/L of each primer, 0.2 mmol/L deoxynucleoside triphosphate, 1.0 U rTaq DNA polymerase (TaKaRa, Dalian, Jinzhou, China), 1 x reaction buffer, and 1.5 mmol/L MgCl_2_. PCR amplification consisted of an initial melting step of 2 min at 95 °C, followed by 35 cycles of 30 s at 94 °C, 30 s at 60 °C for FEN1 –69G/A and 55 °C for FEN1 4150G/T, 30 s at 72 °C, and a final elongation step for 10 min at 72 °C. To distinguish the -69G/A or 4150G/T genotypes, PCR products were subjected to digestion with the restriction enzymes SalI (Sigma Genosys, St. Louis, MO, USA) or Alw26I (Sigma Genosys), respectively. For C.4150 G/T , the PCR product were digested with PvuII (New England Biolabs, Ipswich, MA, USA) overnight at 37 °C. The variant G allele had a PvuII restriction site and after digestion, 2 bands (147 and 112 bp) were generated, while the wild type T allele lacked this restriction site and a single band with a size of 259 bp were obtained. For C.-69G/A, the wild-type allele (A) produced 2 fragment of 112 and 35 bp and the polymorphic allele (G) produce a single 147 bp fragment. To avoid genotyping errors, two researchers independently repeated the genotyping of a limited number of random samples. Confirmation genotyping showed 100% agreement with the original results.

### Statistical analyses

All the analyses were carried out using the Statistical Analysis System software (Version 9.0; SAS Institute, Cary, NC, USA). The PS: Power and Sample Size Calculation program (Vanderbilt School of Medicine, Nashville, TN, USA) were used to determine power and sample size computations according to the methods described in a previously published study^32^. Results of PS analysis indicated that both cancer and control populations were able to provide fair statistical power. In order to match cases and controls in terms of several putative confounding factors (e.g. age and gender), the chi-square and Student’s *t*-tests were used to assess the differences of several qualitative and quantitative traits. To evaluate deviation from the Hardy-Weinberg equilibrium, the discrepancies between observed and expected genotype frequencies in patients and controls were compared by using a chi-square test with one degree of freedom. Allelic association of the SNPs with disease traits was assessed using the Pearson’s 2 × 2 contingency table chi-square test. Gender typical risk of the SNPs for gallbladder cancer, in terms of odds ratio (OR) and 95% confidence interval (CI), was derived from logistic regression models with the SNP genotypes as the explainable variables. Finally, the effect of the FEN1 SNPs and gallstones on gallbladder cancer risk was analyzed using the logistic regression model. The two-locus genotypes, called diplotypes, were defined by the number of risk genotypes at -69G > A and 4150G > T loci. A *p* < 0.05 was considered statistically significant for point-wise statistical analysis. For multiple test analyses, the conservative Bonferroni method was used to correct the *p* value^33^.

## Results

### Characteristics of the study population

Prior to this study, we performed a statistical power analysis using the PS program^33^ to verify that the available cohort was of sufficient size to provide the statistical power necessary for our investigation. Using the population parameter set for effective sample size with an OR of 1.41 and allelic frequency of 0.29, the 341 gallbladder cancer cases and 339 age- and gender-matched healthy controls provided a statistical power of 71.29 and 80.93% at the nominal type I error rate of 0.05 and 0.025, respectively. These results were obtained after performing multiple tests of both SNPs.

Table 1 shows the distribution of age, gender, smoking status, drinking status and gallstone status among cases and controls. The patients and controls were adequately matched in terms of sex and age. The median age was 52.6 years (range, 37–79 years) for the cases and 52.3 years (range, 36–80 years) for the controls (P = 0.82). No significant difference was observed between patients and controls in sex distribution (31.7% males in patients vs.31.3% in controls; P = 0.99). However, smoking incidence, drinking incidence and gallstone incidence were significantly higher in cancer patients than in the control group (*p* < 0.001, P < 0.01, P < 0.001, respectively), and the OR for smoking-associated gallbladder cancer was 12.91 (95% CI, 9.86–26.42), the OR for drinking-associated gallbladder cancer was 9.23 (95% CI, 7.24–17.23), and the gallstone-associated gallbladder cancer was 17.25 (95% CI, 12.41–28.75), suggesting that smoking, drinking and gallstones are important predisposition factors for the development of gallbladder cancer. Of the 341 patients, 54 (15.8%) had pathology grade G1, 81 (23.8%) for grade G2, 162 (47.5) for grade G3 and 44 (12.9) for grade G4. In terms of TNM stage, 31 (9.1%) for 0 stage, 45 (13.2%) for I stage, 44 (12.9%) for II stage, 86 (25.2%) for III stage and 135 (39.6%) for stage IV. In terms of tumor differentiation, 47 (13.8%) patients were classified into the well, 123 (36.%) patients were classified into moderate, 144 (42.2%) patients were classified into poor and 27 (7.9%) were classified into undifferentiated. 202 (59.2%) patients had lymph node metastases, 108 (31.7%) patients had distant metastases, and 146 patients (42.8%) its tumor size were smaller 2 cm (Table 1).

### Association of FEN1 SNPs with gallbladder cancer risk

To determine whether the *FEN1* allele contributed to increased association of gallbladder cancer, we examined the prevalence of *FEN1* alleles in gallbladder cancer cases versus controls. The allelic frequencies of *FEN1* -69A and -4150T were 0.378 and 0.373, respectively, among the 339 healthy controls, and 0.301 and 0.296, respectively, among the 341 gallbladder cancer cases. The genotype frequencies in the control and patient groups conformed to Hardy-Weinberg equilibrium. Linkage disequilibrium analysis showed that *FEN1* -69A and -4150T have strong correlation, with D′ = 0.95 and r^2^ = 0.96. The distribution of *FEN1*-69G > A genotypes among controls(AA, 20.6%; GA, 47.2% and GG 32.2%), the frequencies of the 3 genotypes among gallbladder cancer were AA,11.1%-; GA,48.1%- and GG, 40.8%. The GG genotypes were more prevalent in the cases than in the controls (P < 0.001). Significantly increased association with gallbladder cancer was observed for subjects with both the *FEN1*-69G > A GA (OR = 1.73, 95% CI = 1.02–2.63) and the *FEN1*-69G > A GG (OR = 2.29, 95% CI = 1.31–3.95) (Table 2). This association was not affected by adjusting other factors (age, sex and gallstone) via regression analysis (OR_adjust_ = 1.74, 95% CI = 1.02–2.64 for the *FEN1*-69G > A GA genotypes; OR_adjust_ = 2.31, 95% CI = 1.32–3.96 for the *FEN1*-69G > A GG genotypes). In the meantime, the distribution of *FEN1* -4150T genotypes among controls(TT, 21.8%;GT, 49.3% and GG 28.9%), the frequencies of the 3 genotypes among gallbladder cancer were TT,12.9%-; GT, 48.4% and GG 38.7%. The GG genotypes was also more prevalent in the cases than in the controls (P < 0.001). Significantly increased association for gallbladder cancer was observed for subjects with both the *FEN1*-4150T GT(OR = 1.93, 95% CI = 1.04–2.91) and the *FEN1*-4150T GG(OR = 2.56, 95% CI = 1.37–5.39) (Table 2). These results was not affected by adjusting other factors (age, sex and gallstone) via regression analysis (OR_adjust_ = 1.95, 95% CI = 1.09–2.94 for the *FEN1*-4150T GT genotype; OR_adjust_ = 2.57, 95% CI = 1.39–5.42 for the *FEN1*-4150T GG genotype). These results where consistent with the fact that a significantly trend towards increased association was observed with predicted less protective *FEN1* genotypes (P < 0.001, χ^2^ trend test, Table 2). Although stratification analyses by age, sex, smoking status or drinking status were also conducted, no further evidence was observed (data not shown). In this study, FEN1 -69GA and 4150GT SNPs were not associated with other clinicopathological parameters, such as age, sex, pathology grade , TNM stage , tumor differentiation , lymph node metastasis , size or metastasis of tumors (Table 3).

### The relationship between FEN1 genotype gallbladder cancer association by exposure to gallstone

To determine the relationship between FEN1 genotype and gallbladder cancer by exposure to gallstone, we stratified study subjects by FEN1 genotype and gallstone status (Table 2). We observed no statistically significant association between FEN1 genotypes and gallbladder cancer in gallstone absence. In contrast, near-significant increases in risk for gallbladder cancer were observed for gallstone presence with the *FEN1*-69G > A GA (OR = 2.3, 95% CI = 1.5–3.5) and the *FEN14150G* > *T* GT (OR = 3.7, 95% CI = 1.4–9.8), whereas significant increases in association for gallbladder cancer were observed for gallstone presence with the *FEN1*-69G > A GG (OR = 6.8, 95% CI = 2.1–28.31) and the *FEN14150G* > *T* GG(OR = 7.2, 95% CI = 2.2–20.2). These data corresponded with the fact that a significant trend towards increased association for gallbladder cancer was observed with potentially higher-risk *FEN1*-69G > A genotypes (P < 0.001, χ^2^ trend test) and *FEN14150G* > *T* (P < 0.001, χ^2^ trend test) in gallstone presence but not in gallstone absence(P = 0.81, P = 0.89, respectively). A significant increase in risk for gallbladder cancer was observed for larger gallstone (those with stone diameters 2 cm or greater) with the *FEN1*-69G > A GA (OR = 2.1, 95% CI = 1.0–4.5) and the *FEN14150G* > *T* GT (OR = 2.3, 95% CI = 1.0–4.9), whereas significant increases in association for gallbladder cancer were also observed for larger gallstone (those with stone diameters 2 cm or greater) with the *FEN1*-69G > A GG (OR = 7.3, 95% CI = 1.8–18.8) and the *FEN14150G* > *T* GG (OR = 8.2, 95% CI = 1.5–19.8). These data are consistent with the observation that a significant increase trend risk for gallbladder cancer was obvious with potentially higher-risk *FEN1*-69G > A genotypes and *FEN14150G* > *T* genotypes in gallbladder cancer patients with larger gallstone(P < 0.001, P < 0.001, respectively, χ^2^ trend test). In the meantime, we observed a statistically significant association between *FEN1*genotype and gallbladder cancer risk in gallbladder cancer patients with smaller gallstone (those with stone diameters 2 cm smaller) (FEN1-69G > A GA:OR = 2.0, 95% = 0.7–3.9; FEN1-69G > A GG:OR = 3.5, 95% = 1.7–17.2; χ^2^ = 13.4, p < 0.001; FEN14150G > T GT:OR = 2.2, 95% = 0.8–4.3; FEN14150G > T GG:OR = 5.5, 95% = 1.3–16.7,χ^2^  = 13.7, P < 0.001).

### Association of FEN1 haplotypes with gallbladder cancer risk

Haplotype analyses showed that the *FEN1* A-69G4150, G-69G4150 and G-69T4150 haplotypes were associated with a significantly increased risk of gallbladder cancer. The adjusted ORs were 1.29 (95% CI = 1.11–1.52, *p* = 0.032); 2.14 (95% CI = 1.28–3.38, *p* = 0.023); 2.79 (95% = 1.94–3.99, *P* = 0.0009), respectively, when compared to the *FEN1* A-69T4150 haplotype (Table 4).

## Discussion

To the best of our knowledge, the current study is the first to assess the risk of gallbladder cancer associated with the *FEN1* -69G > A and -4150G > T SNPs in a Chinese population. In this population, we found a significantly increased gallbladder cancer risk among carriers of the FEN1 -69G and 4150G alleles and the G-69G4150 haplotype compared with carriers of the -69A and 4150T alleles, the G-69T4150 T alleles or the G-69T4150 haplotype. Moreover, diplotypes and genotypic dosage was also associated with a significantly elevated risk when compared with the risk associated with individual loci. Our findings raised the possibility that the two loci may interact with gallstones to synergistically increase gallbladder cancer risk. Our data confirmed that *FEN1* polymorphisms and haplotypes were associated with elevated gallbladder cancer risk, and that gallstones synergistically increased this gallbladder cancer risk.

DNA repair enzyme maintenance of genomic integrity is an essential component of normal cell homeostasis, and is necessary to maintain cell growth, differentiation and apoptosis^34^,^35^. Evidence increasingly indicates that polymorphisms in human DNA repair genes alter DNA repair capacity and are associated with increased solid tumor risk and susceptibility^23^,^24^. FEN1 is expressed in many species, from archaebacteria to humans, and FEN1 functional deficiency may lead to genomic instability and cancer development^18^. One example illustrating the importance of the anti-cancer role of FEN1 was demonstrated using *FEN1* knockout mice. While the homozygous *FEN1* knockout was embryonically lethal, FEN1 heterozygous mice were viable and appeared to be healthy^19^. However, FEN1 heterozygous knockout mice that were also heterozygous for an *APC* gene mutation had increased cancer development and reduced survival^36^, indicating that FEN1 may function as a tumor suppressor gene^14^,^37^,^38^. Therefore, we predict that decreased FEN1 expression or altered FEN1 function could result in the malignant transformation of normal cells^39^ or increase the susceptibility of patients to other carcinogens or environmental factors^17^. Mechanistically, FEN1 mutations could induce single-stranded DNA breaks and the subsequent collapse of DNA replication forks, leading to DNA replication stress^17^. Polyploidy in cancer cells could lead to the overexpression of BRCA1, p19^arf^ and other DNA repair genes in FEN1 mutant cells. This overexpression could trigger the single-stranded DNA break repair and non-homologous end-joining pathways, increasing DNA repair activity at the cost of frequent chromosomal translocations^40^. Our published^28^,^29^ and current data support the hypothesis that genetic variants that influence DNA repair capacity play an important role in human tumorigenesis.

No single causative factor has yet been identified for gallbladder cancer, but a number of predisposing and putative etiologies have been associated with altered DNA replication^13^,^40^. These include cholelithiasis, gallbladder polypoidal lesions, genetic predisposition, chemical carcinogens, anatomical variations of the pancreaticobiliary ductal system, infected bile, carrier-state typhoid fever, and ulcerative colitis^41^,^42^,^43^. In this study, we found significantly increased gallbladder cancer risk among carriers of the FEN1 -69G and 4150G alleles and the G-69G4150 haplotype when compared with carriers of the -69A and 4150T alleles, the G-69T4150 T alleles or the G-69T4150 haplotype. These results are consistent with the findings, while other types indicate that of previous studies^25^,^26^,^27^, indicating that these genetic variants may be common cancer risk factors.

Gallstones are an established risk factor for gallbladder cancer^44^,^45^, the reported prevalence of gallstones in patients with gallbladder cancer ranges from 74% to 92% in European countries, whereas, in the U.S., gallstone prevalence is estimated to be 10% in the general population^41^. In China, a review of 3922 cases showed that the 49.7% of gallbladder cancer cases had concurrent gallstones^43^, while gallstone prevalence in the general Chinese population was estimated to be 7.2% (7,023/105,019). In previous clinical and population-based studies in China, inflammatory processes associated with gallstone and cholecystitis have been linked to the development of gallbladder cancer, gallstones are associated with an 18-fold risk of gallbladder cancer, and the combination of gallstones and cholecystitis increases the risk of gallbladder cancer by 34-fold^45^. In this study, we found that 57.8% of gallbladder cancer patients had concurrent gallstones (197 of 341). This result was significantly higher than the percentage of cholelithiasis observed in the healthy control group (6.2%, 21 of 339). Additionally, physical trauma caused by gallstones or bile-containing carcinogens may synergistically induce epithelial dysplasia, encouraging the ultimate progression to carcinoma^46^,^47^. Our previous study demonstrated that cholelithiasis and cholecystitis produced a series of pathological epithelial changes, including simple epithelial hyperplasia, atypical hyperplasia and carcinoma in situ^47^. These pathological changes are all considered precancerous lesions of gallbladder carcinoma^48^,^49^. The current study demonstrated that the interaction of genetic factors and the environment, in this case the interaction of *FEN1* polymorphisms and gallstones, could synergistically increase the risk of gallbladder cancer.

## Potential Study Limitations

Our study has some limitations. First, since this study is a hospital-based case and control study, gallbladder cancer cases and controls from the hospital may have an inherent selection bias. Thus, a population-based prospective study is needed to validate our data. Second, only small number of the healthy controls in this study had gallstones. Therefore, the complicated genetic models of the *FEN1* genotypes/diplotypes for gallbladder cancer could not be accurately matched with controls defined by the presence of gallstones or gallstones of varying size. Third, detection of both gene-gene and gene-environment interactions often requires a very large sample size; thus, the limited sample size of this study may not provide sufficient statistical power to explore these synergic effects. Significant interactions between FEN1 -69G > A (GA or GG) and 4150G > T (GT or GG) or between these genetic factors and cholelithiasis were identified. However, further investigation is needed in large and independent ethnic populations.

## Additional Information

**How to cite this article**: Jiao, X. *et al*. Variants and haplotypes in Flap endonuclease 1 and risk of gallbladder cancer and gallstones : a population-based study in China. *Sci. Rep*. **5**, 18160; doi: 10.1038/srep18160 (2015).

## Figures and Tables

**Table 1:** Distribution of selected characteristics among gallbladder cancer patients and controls.

Variable	Controls n (%) (N = 339)	Casesn (%) (N = 341)	P^a^
Sex			
Male	106 (31.3)	108 (31.7)	P = 0.99
Female	233 (68.7)	233 (68.3)	
Age(years)			
<55	203 (59.9)	204 (59.8)	P = 0.82
55–64	69 (20.3)	72 (21.1)	
≥65	67 (19.8)	65 (19.1)	
Smoking status			
No	162 (47.8)	119 (34.9)	P < 0.001
Yes	177 (52.2)	222 (65.1)	
Drinking status			
No	278 (82.0)	249 (73.0)	P < 0.01
Yes	61 (18.0)	92 (27.0)	
Gallstone status, n(%)			
Absence	318 (93.8)	144 (42.2)	P < 0.001
Presence	21 (6.2)	197 (57.8)	
Size of largest gallstone			
<2 cm	9 (42.9)	83 (42.1)	P = 0.97
≥2 cm	12 (57.1)	114 (57.9)	
Pathology grade			
G1	-	54 (15.8)	
G2	-	81 (23.8)	
G3	-	162 (47.5)	
G4	-	44 (12.9)	
TNM stage			
0	-	31 (9.1)	
I	-	45 (13.2)	
II	-	44 (12.9)	
III	-	86 (25.2)	
IV	-	135 (39.6)	
Tumor differentiation			
Well	-	47 (13.8)	
Moderate	-	123 (36.1)	
Poor	-	144 (42.2)	
Undifferentiated	-	27 (7.9)	
Lymph node metastases			
+	-	202 (59.2)	
−	-	139 (40.8)	
Distant metastases			
+	-	108 (31.7)	
−	-	233 (68.3)	
Tumor size			
<2 cm	-	146 (42.8)	
≥2 cm	-	195 (57.2)	

^a^Two-sided χ^2^ test.

**Table 2:** Prevalence of FEN1 genotype frequencies and gallbladder cancer risk by gallbladder stone.

	FEN1 genotype	Controls n(%)	Cases n(%)	Crude OR 95% CI)	Adjusted OR^a^ (95% CI)	Trend test (P value)^b^
Total	C.-69G > A					
	AA	70 (20.6)	38 (11.1)^c^	1.0 (reference)	1.0 (reference)	P < 0.001
	GA	160 (47.2)	164 (48.1)	1.73 (1.02–2.63)	1.74 (1.02–2.64)	
	GG	109 (32.2)	139 (40.8)	2.29 (1.31–3.95)	2.31 (1.32–3.96)	
Sex						
Male	AA	22 (20.8)	12 (11.1)	1.0 (reference)	1.0 (reference)	P < 0.001
	GA	50 (47.2)	51 (47.3)	1.75 (1.03–2.65)	1.76 (1.04–2.66)	
	GG	34 (32.0)	45 (42.6)	2.42 (1.32–4.96)	2.43 (1.35–4.98)	
Female	AA	48 (20.6)	26 (11.2)	1.0 (reference)	1.0 (reference)	P < 0.004
	GA	110 (47.2)	113 (48.5)	1.63 (1.01–2.58)	1.64 (1.02–2.59)	
	GG	75 (32.2)	94 (40.3)	2.18 (1.29–3.37)	2.19 (1.30–3.38)	
Gallstone status						
Absence	AA	67 (21.1)	30 (20.8)	1.0 (reference)	1.0 (reference)	P = 0.81
	GA	145 (45.6)	66 (45.8)	1.1 (0.3–3.0)	1.1 (0.3–3.1)	
	GG	106 (33.3)	48 (33.3)	1.2 (0.4–3.2)	1.3 (0.4–3.3)	
Presence	AA	3 (14.3)	8 (4.1)	1.0 (reference)	1.0 (reference)	P < 0.001
	GA	15 (71.4)	98 (49.7)	2.2 (1.4–3.4)	2.3(1.5–3.5)	
	GG	3 (14.3)	91 (46.2)	5.9 (1.8–18.6)	6.8 (2.1–28.3)	
Size of largest gallstone						
<2 cm	AA	3 (33.3)	3 (3.6)	1.0 (reference)	1.0 (reference)	P < 0.001
	GA	6 (66.7)	57 (68.7)	1.9 (0.7–3.8)	2.0 (0.7–3.9)	
	GG	0 (0)	23 (27.7)	3.4 (1.6–16.2)	3.5 (1.7–17.2)	
≥2 cm	AA	0 (0)	5 (4.4)	1.0 (reference)	1.0 (reference)	P < 0.001
	GA	9 (75.0)	41 (36.0)	2.0 (0.9–4.2)	2.1 (1.0–4.5)	
	GG	3 (25.0)	68 (59.6)	7.2 (1.7–18.6)	7.3 (1.8–18.8)	
Total	C.4150G > T					
	TT	74 (21.8)	44 (12.9)^d^	1.0 (reference)	1.0 (reference)	P < 0.001
	GT	167 (49.3)	166 (48.4)	1.93 (1.04–2.91)	1.95 (1.09–2.94)	
	GG	98 (28.9)	131 (38.7)	2.56 (1.37–5.39)	2.57 (1.39–5.52)	
Sex						
Male	TT	23 (21.7)	14 (13.0)	1.0 (reference)	1.0 (reference)	P < 0.001
	GT	52 (49.1)	52 (48.1)	1.59 (1.01–2.32)	1.60 (1.02–2.33)	
	GG	31 (29.2)	42 (38.9)	2.43 (1.31–3.42)	2.44 (1.32–3.43)	
Female	TT	51 (21.9)	30 (12.5)	1.0 (reference)	1.0 (reference)	χ^2^ < 0.001
	GT	114 (48.9)	114 (48.9)	1.71 (1.01–2.62)	1.72 (1.02–2.63)	
	GG	68 (29.2)	89 (38.6)	2.16 (1.22–4.91)	2.17 (1.23–4.92)	
Gallstone status						
Absence	TT	69 (21.8)	29 (20.1)	1.0 (reference)	1.0 (reference)	P = 0.89
	GT	152 (47.8)	68 (47.2)	1.2 (0.4–3.1)	1.3 (0.5–3.2)	
	GG	97 (30.4)	47 (32.6)	1.3 (0.5–3.8)	1.4 (0.6–3.9)	
Presence	TT	3 (14.3)	15 (7.6)	1.0 (reference)	1.0 (reference)	P < 0.001
	GT	15 (71.4)	98 (49.7)	3.6 (1.4–9.6)	3.7 (1.4–9.8)	
	GG	3 (14.3)	84 (42.6)	7.1 (2.1–20.1)	7.2 (2.2–20.2)	
Size of largest gallstone						
<2 cm	TT	3 (33.3)	3 (3.6)	1.0 (reference)	1.0 (reference)	P < 0.001
	GT	6 (66.7)	55 (66.3)	2.1 (0.8–4.2)	2.2 (0.8–4.3)	
	GG	0 (0)	25 (30.1	5.4 (1.2–16.6)	5.5 (1.3–16.7)	
≥2 cm	TT	0 (0)	12 (10.5)	1.0 (reference)	1.0 (reference)	P < 0.001
	GT	9 (75.0)	43 (37.7)	2.2 (0.9–4.8)	2.3 (1.0–4.9)	
	GG	3 (25.0)	59 (51.8)	8.1 (1.4–19.4)	8.2 (1.5–19.8)	

^a^Adjusted for age, sex and gallstone.

^b^Trend test assessing correlation between gallbladder cancer risk and predicting high risk FEN1 genotypes.

^c^Numbers in parenthesis refer to percentages.

^d^Numbers in parenthesis refer to percentages.

**Table 3:** Association of genotypic data with clinicopathological features of gallbladder carcinoma patients.

Variable (# of cases)	C.69G > A						C.4150G > T					
	AA	GA	GA	Crude OR (95%)	Adjust OR (95% CI)^a^	Trend test (P value)^b^	TT	GT	GG	Crude OR (95%)	Adjust OR (95% CI)^a^	Trend test (P value)^b^
Sex						P = 0.83						P = 0.97
Male	12	51	45	1.0 (reference)	1.0 (reference)		14	52	42	1.0 (reference)	1.0 (reference)	
Female	26	113	94	1.1 (0.2–2.1)	1.1 (0.3–2.2)		30	114	89	1.05 (0.1–1.1)	1.06 (0.2–1.2)	
Pathology grade						P = 1.0						P = 1.0
G1 (54)	6	25	23				7	27	20			
G2 (81)	9	37	35				10	39	32			
G3 (162)	18	81	63				21	81	60			
G4 (44)	5	21	18				6	18	20			
TNM stage						P = 1.0						P = 1.0
I (31)	4	15	12				3	15	13			
II (45)	5	24	16				6	25	14			
III (44)	5	21	18				5	20	19			
IV (86)	9	40	37				10	41	35			
V (135)	15	64	56				20	64	51			
Tumor differentiation						P = 1.0						P = 1.0
Well (47)	6	25	16				6	25	16			
Moderate (123)	13	58	52				15	59	49			
Poor (144)	16	70	58				17	69	58			
Undifferentiated (27)	3	11	13				6	12	9			
Lymph node metastasis						P = 1.0						P = 1.0
+(202)	22	95	85				25	97	80			
−(139)	16	69	54				19	68	52			
Distant metastasis						P = 1.0						P = 1.0
+(108)	12	52	44				14	54	40			
−(233)	26	112	95				30	111	92			
Tumor size						P = 1.0						P = 1.0
<2 cm (146)	17	74	55				20	70	56			
≥2 cm (195)	21	90	84				24	95	76			

^a^Adjusted for age, sex, pathology grade , TNM stage , tumor differentiation , lymph node metastasis , distant metastasis , tumor size.

^b^Trend test assessing correlation between gallbladder cancer risk and predicting high risk FEN1 genotypes.

**Table 4:** Association of *FEN1* haplotypes with gallbladder cancer risk.

Haplotype	Chromosome number		OR^a^ (95% CI)	*p*-value^b^
	Case n (%) (n = 341)	Control n (%) (n = 339)		
A-69T4150	94 (27.6)	124 (36.7)	1.00 (Reference)	
A-69G4150	218 (63.9)	206 (60.8)	1.29 (1.1–1.5)	0.032
G-69G4150	8 (2.3)	4 (1.1)	2.14 (1.2–3.3)	0.023
G-69T4150	21 (6.2)	5 (1.4)	2.79 (1.9–3.9)	0.0009

OR, odds ratio; CI confidence interval.

^a^Adjusted for sex, age and gallstone status.

^b^After 1000 permutation tests.
